# Gender-specific change in leptin concentrations during long-term CPAP therapy

**DOI:** 10.1007/s11325-019-01846-y

**Published:** 2019-05-04

**Authors:** Miia Aro, Ulla Anttalainen, Samu Kurki, Kerttu Irjala, Olli Polo, Tarja Saaresranta

**Affiliations:** 1 Division of Medicine, Department of Pulmonary Diseases, Turku University Hospital, PO Box 52, FI-20520 Turku, Finland; 2 Department of Pulmonary Diseases and Clinical Allergology, University of Turku, Turku, Finland; 3 Sleep Research Centre, Department of Pulmonary Diseases and Clinical Allergology, University of Turku, Turku, Finland; 4 Auria Biobank, University of Turku and Turku University Hospital, Turku, Finland; 5 Department of Clinical Chemistry, University of Turku, Turku, Finland; 6 Department of Pulmonary Diseases, Tampere University Hospital, Tampere, Finland

**Keywords:** Sleep apnoea, Leptin, Long-term CPAP therapy, Gender-specific

## Abstract

**Purpose:**

Nasal continuous positive airway pressure (CPAP) alleviates sleepiness in patients with obstructive sleep apnoea syndrome (OSAS), but part of OSAS patients keep gaining weight. Leptin and insulin-like growth factor-1 (IGF-1) interact with energy balance, and CPAP therapy has been suggested to influence these endocrine factors. We hypothesised that leptin would decrease during long-term CPAP therapy, and weight gain would associate with OSAS severity, lower CPAP adherence, lower IGF-1, and leptin concentrations.

**Methods:**

Consecutive patients (*n* = 223) referred to sleep study with suspected OSAS were enrolled. Patients underwent cardiorespiratory polygraphy at baseline. Questionnaires were completed, and blood samples were drawn both at baseline and after 3 years. A total of 149 (67%; M 65, F 84) patients completed the follow-up. Plasma samples were available from 114 patients, 109 of which with CPAP adherence data (49 CPAP users, 60 non-users).

**Results:**

At baseline, the CPAP users were more obese and had more severe OSAS than the non-users. Leptin concentrations did not differ. After follow-up, leptin concentrations were higher in CPAP users (30.2 ng/ml vs. 16.8 ng/ml; *p =* 0.001). In regression analysis, increase in leptin concentrations was independent of age, baseline body mass index (BMI), or the change in BMI. Leptin concentrations increased among females (− 8.9 vs. 12.7 ng/ml; *p* < 0.001); whereas in men, CPAP did not have an effect, if not opposed the natural decrease in leptin observed in men not using CPAP. Change in IGF-1 levels did not differ.

**Conclusions:**

Our results suggest increase in leptin concentrations during long-term CPAP therapy among females.

**Electronic supplementary material:**

The online version of this article (10.1007/s11325-019-01846-y) contains supplementary material, which is available to authorized users.

## Introduction

Obesity is a major risk factor for obstructive sleep apnoea syndrome (OSAS), over 70% of patients with OSAS being obese [1]. The recent prevalence estimates of OSAS in the Western societies are as high as 17% due to increased obesity [2]. Nasal continuous positive airway pressure (CPAP) therapy is a treatment of choice for OSAS [3]. The regulation of energy balance in OSAS is multifactorial, and the data on the effect of CPAP therapy on energy balance is limited. CPAP therapy effectively reduces daytime sleepiness [4], which in turn could help to increase physical activity and to choose healthier food. However, proper treatment with nasal CPAP seems not to promote weight loss, especially in females [5] [6].

Endocrine factors such as leptin [7] or insulin-like growth factor-1 (IGF-1) [8] have been linked with energy balance. Central obesity is composed of adipose tissue, which is a major source of satiety hormone leptin, that plays an important role in metabolic control, reproduction, and neuroendocrine signalling [7]. Both leptin and IGF-1 concentrations have been reported to decrease during short-term CPAP treatment [9], which should favour weight gain. On the other hand, obese people have increased leptin concentrations indicating obesity-linked leptin-resistance [10]. It has also been demonstrated, that patients with OSAS have even higher leptin concentrations than the age- and BMI-matched controls [11], although this is not confirmed in all studies [12]. Leptin resistance may lead to increased caloric intake and make weight management difficult for these patients [13]. However, intermittent hypoxemia and sleep fragmentation may stimulate leptin secretion, and therefore, leptin might prevent respiratory depression in obesity [14], which could in turn explain increased leptin concentrations among patients with OSAS. Effective treatment of OSAS has been suggested to reduce leptin concentrations by changing blood flow in the body via haemodynamic changes that increase lung volume, abdominal pressure, or visceral blood flow, resulting in increased oxygen supply to tissue, thereby reducing leptin concentrations [15].

We hypothesised that weight gain in patients, referred for OSAS evaluation, is linked with more severe OSAS, lower compliance to CPAP therapy, and lower leptin and IGF-1 concentrations. Further, we hypothesised that leptin concentrations in CPAP users would decrease during long-term follow-up, as previously shown during short-term CPAP therapy [9].

## Methods

Data were prospectively collected in consecutive patients, who were referred to the Department of Pulmonary Diseases at Turku University Hospital with suspected OSAS, from March 2004 to October 2006. The original cohort comprised of 223 patients, involving 101 men (45.3%) and 122 women (54.7%). The study protocol was approved by the Ethics Committee of the Hospital District of Southwest Finland, Turku, Finland. All patients gave their written informed consent.

All the patients came to the hospital the evening prior to the overnight cardiorespiratory polygraphy. A nurse measured their weight and height. Body mass index (BMI) was calculated as weight in kilograms divided by the square of height in meters (kg/m^2^). Excessive daytime sleepiness was assessed with the help of the Epworth Sleepiness Scale (ESS) [16]. ESS score (range 0–24) over 10 points was considered abnormal. Self-reported usual sleep duration and sleep timing were recorded. Medication was recorded by the Anatomic Therapeutic Chemical Classification (ATC) system. Smoking was asked with a question: “Have you ever smoked 6 months or more during your life?”. Choices were (1) yes or (2) no. Alcohol consumption was categorised as (1) not at all (2) 1–6 alcohol doses per week, (3) 7–14 alcohol doses per week, or (4) 15–24 alcohol doses per week.

Venous blood was drawn after an overnight fast to measure leptin, IGF-1, and fasting blood glucose concentrations. Blood samples were stored in ice and centrifuged immediately, and then kept frozen in − 70 °C until analysed. Leptin was assayed with the ELISA method and IGF-1 with immunoluminometric assay (DRG Instruments GmbH®, Marburg, Germany).

Patients underwent a complete overnight in-hospital cardiorespiratory polygraphy (Embla®, Medcare Flaga hf, Medical Devices, Reykjavik, Iceland), which included measurements of inspiratory flow pressure profile via nasal prongs, abdominal and thoracic movements, electrocardiography, periodic leg movements, sleeping position, transcutaneous carbon dioxide partial pressure (PTcCO_2_; TCM3, Radiometer A/S, Copenhagen, Denmark), and arterial oxyhaemoglobin saturation (SaO_2_). SaO_2_ was measured with a finger probe pulse oximeter (Oximeter Embla A10 XN, Embla, Denver, CO, USA). The episodes of arterial oxyhaemoglobin desaturation of 4% units or more per hour (oxygen desaturation index, ODI4) were automatically determined from the SaO_2_ signals with Somnologica software. Possible artefacts were manually removed, and episodes of apnoea and hypopnoea were visually determined by an experienced scorer and expressed per hour (apnoea–hypopnea index, AHI) in bed from lights off to lights on, using internationally accepted criteria [17]. Respiratory effort-related arousals (RERA) were not scored since an electroencephalogram was not included in the set-up.

All the patients had symptoms suggesting for OSAS, which was diagnosed if AHI was ≥ 5 per h. Patients, whose AHI was over 15 per h, were introduced to CPAP therapy. Moreover, if the patients suffered from severe symptoms, CPAP therapy was also commenced with AHI 5–15 per h. All the patients with CPAP therapy had a follow-up visit 3 months after the initiation, and then once every year.

After 3 years, all the patients from the original cohort were invited for a follow-up visit; when the same measurements, except cardiorespiratory polygraphy, were repeated, their CPAP pressure was checked, and average hours of use were documented with within-built clock counters.

**Fig. 1:**
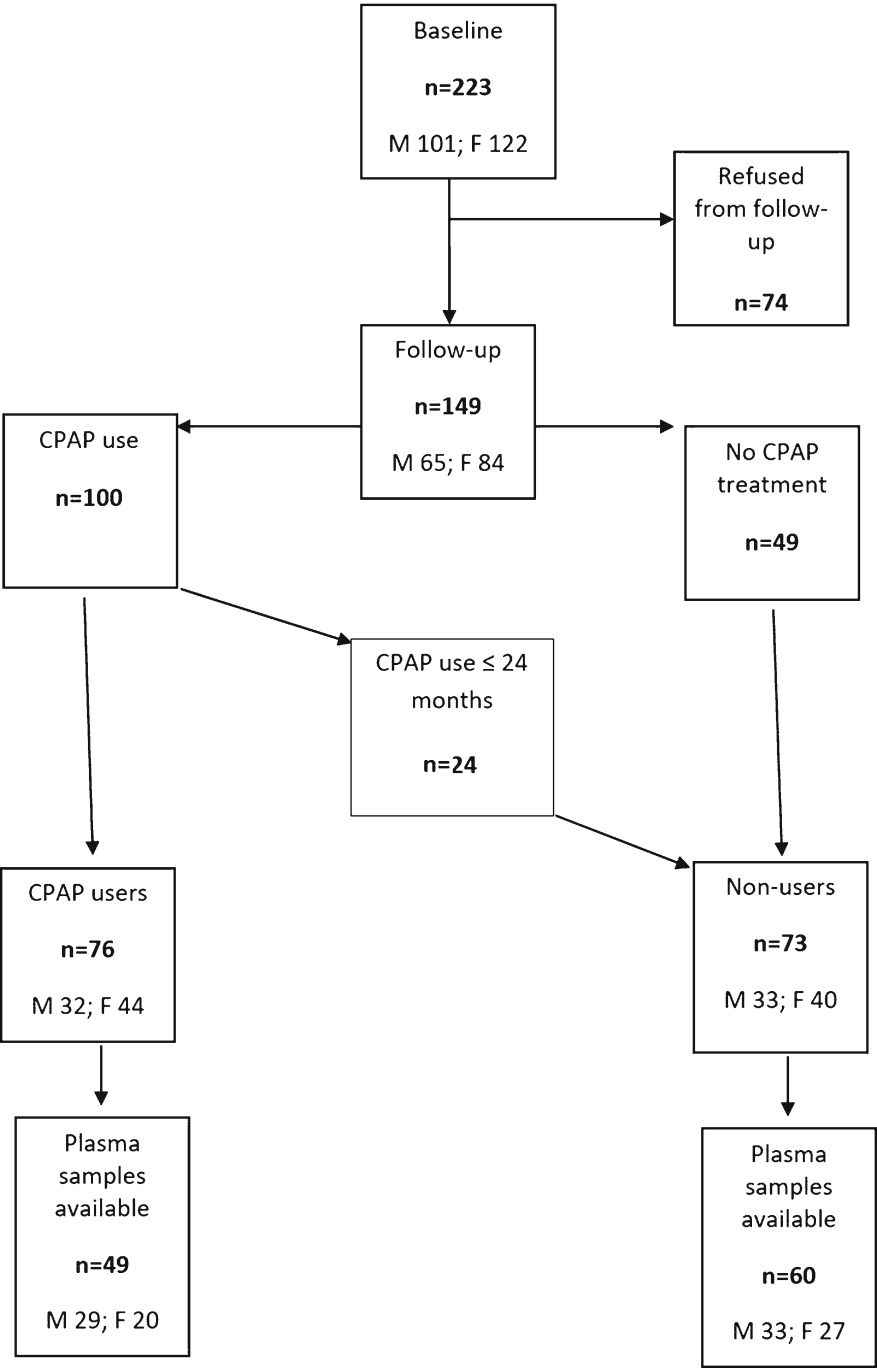
Flow-chart of patients included in the study

After the follow-up period, the original cohort was divided into the CPAP users and the non-users (Fig. 1). “User” was defined as a patient who still used regularly CPAP after 3 years. Those who had discontinued their CPAP use before the 3-year follow-up, were considered “non-users”. At the 3-year follow-up, 149 (66.8%; M 65, F 84) of the patients participated. Of the 149, 76 patients (51.0%; M 32, F 44) used CPAP. Among the non-users, there were 49 patients who refused CPAP treatment, seven patients who used it less than 3 months, five patients for 3–11 months, and nine for 12–24 months. From the remaining patients, both baseline and follow-up leptin and IGF-1 concentrations were available from 114 patients, and they were included in the study. Forty-nine of them (43.0%; M 29, F 20) were CPAP users and 60 were non-users (52.6%; M 33, F 27). The data of CPAP use was not found from five of these patients (4.4%; M 3, F 2). The data of average hours of CPAP use were available from 48 patients (M 29, F 19).

### Data analyses

Data are presented as median with interquartile range or mean with range. The limit of quantitation was 1.00 ng/ml in leptin, and 3.25 nmol/l in IGF-1. Therefore, the leptin concentrations < 1.00 ng/ml were imputed with 0.50 ng/ml, and IGF-1 < 3.25 ng/ml with a value of 1.63 ng/ml.

To study the effect of covariates on the within-patient 3-year change in leptin concentrations, an ordinary least squares linear regression model was fitted for both genders. Conditional inference trees were used to find and visualise statistically significant interactions between categorical covariates. Bipartite correlations between variables were studied with Spearman’s rank correlation coefficients. To compare groups, Mann–Whitney *U* test was used for continuous variables and chi-squared test for categorical variables. *p* values less than 0.05 were considered statistically significant, and all *p* values were two-sided. Statistical analyses were performed with IBM SPSS version 23 (IBM Corp. Released 2015. IBM SPSS Statistics for Windows, Version 23.0. Armonk, NY: IBM Corp).

## Results

### Baseline

The baseline characteristics of the study population are presented in Table 1. At baseline, the CPAP users had higher BMI (*p* < 0.001), AHI (*p* < 0.001), and ODI4 (*p* < 0.001) compared with the non-users (Table 2). They also had lower mean (*p* < 0.001) and minimum (*p* = 0.001) arterial oxyhemoglobin saturation. The leptin concentrations did not differ. When males and females were evaluated separately, there were no differences at baseline (male 12.4 ng/ml vs. female 18.0 ng/ml, *p* = 0.578) (Table 1).

**Table 1:** Baseline characteristics of the patients (*n* = 114)

	Male *n* = 65 (57%)		Female *n* = 49 (43%)		*p* value
	Median	IQR (25–75%)	Median	IQR (25–75%)	
Age (years)	55.0	48.0–61.0	54.5	45.8–61.0	0.997
BMI (kg/m^2^)	30.2	27.3–34.3	32.0	27.2–36.0	0.277
AHI (no./h)	14.2	6.0–19.9	10.5	4.9–17.7	0.591
ODI_4_ (no./h)	5.9	2.8–12.8	6.8	2.3–12.7	0.434
Mean SaO_2_ (%)	94.5	92.7–95.5	94.7	93.0–95.9	0.403
Min SaO_2_ (%)	86.0	82.0–88.0	85.5	79.8–87.0	0.224
Leptin (ng/ml)	12.4	7.6–29.1	18.0	7.9–34.5	0.578
IGF-1 (nmol/l)	14.0	11.2–18.5	11.1	8.2–14.8	0.071

Values are presented as median (interquartile range). *BMI* body mass index, *AHI* apnoea–hypopnoea index, *ODI*_*4*_ oxygen desaturation index, *SaO*_*2*_ oxyhaemoglobin saturation, *IGF-1* insulin-like growth factor-1

**Table 2:** Differences between the CPAP users and the non-users (*n* = 109)

	CPAP users (*n* = 49)		Non-users (*n* = 60)		
	Median	IQR (25–75%)	Median	IQR (25–75%)	*p* value
Age	56.00	45.00–61.00	53.00	47.00–59.00	0.550
Baseline BMI (kg/m^2^)	33.70	30.90–37.08	28.30	26.15–33.65	*< 0.001*
Follow-up BMI (kg/m^2^)	34.73	30.62–39.36	29.05	26.08–32.34	*0.001*
Change in BMI (kg/m^2^)	0.35	− 0.63–2.77	− 0.03	− 1.49–0.63	0.250
AHI baseline (#/h)	18.05	11.70–49.55	8.69	4.09–14.50	*< 0.001*
ODI_4_ baseline (*#/*h)	11.70	4.57–38.90	3.90	1.50–8.05	*< 0.001*
Mean SaO_2_ baseline (%)	93.60	91.95–94.98	95.00	93.70–96.20	*< 0.001*
Min SaO_2_ baseline (%)	83.00	77.50–86.00	87.00	83.00–89.00	*0.001*
Leptin baseline (ng/ml)	17.25	7.98–33.75	16.70	7.85–35.39	0.870
Leptin follow-up (ng/ml)	26.95	8.65–48.09	8.60	4.45–23.69	*0.002*
Change in leptin (ng/ml)	9.13	− 7.82–29.72	− 5.86	− 22.93–8.29	*0.007*
Baseline IGF-1 (nmol/l)	13.60	9.08–17.78	11.8	9.11–17.15	0.594
Follow-up IGF-1 (nmol/l)	15.65	12.20–19.00	15.70	12.15–19.70	0.933
Change in IGF-1 (nmol/l)	2.60	− 3.79–7.53	3.20	− 2.75–7.70	0.775

Values are presented as median (interquartile range). *BMI* body mass index, *AHI* apnoea–hypopnoea index, *ODI*_*4*_ oxygen desaturation index, *SaO*_*2*_ arterial oxyhaemoglobin saturation, *IGF-1* insulin-like growth factor-1, Statistically significant values are shown in italics

### Three-year follow-up

**Fig. 2:**
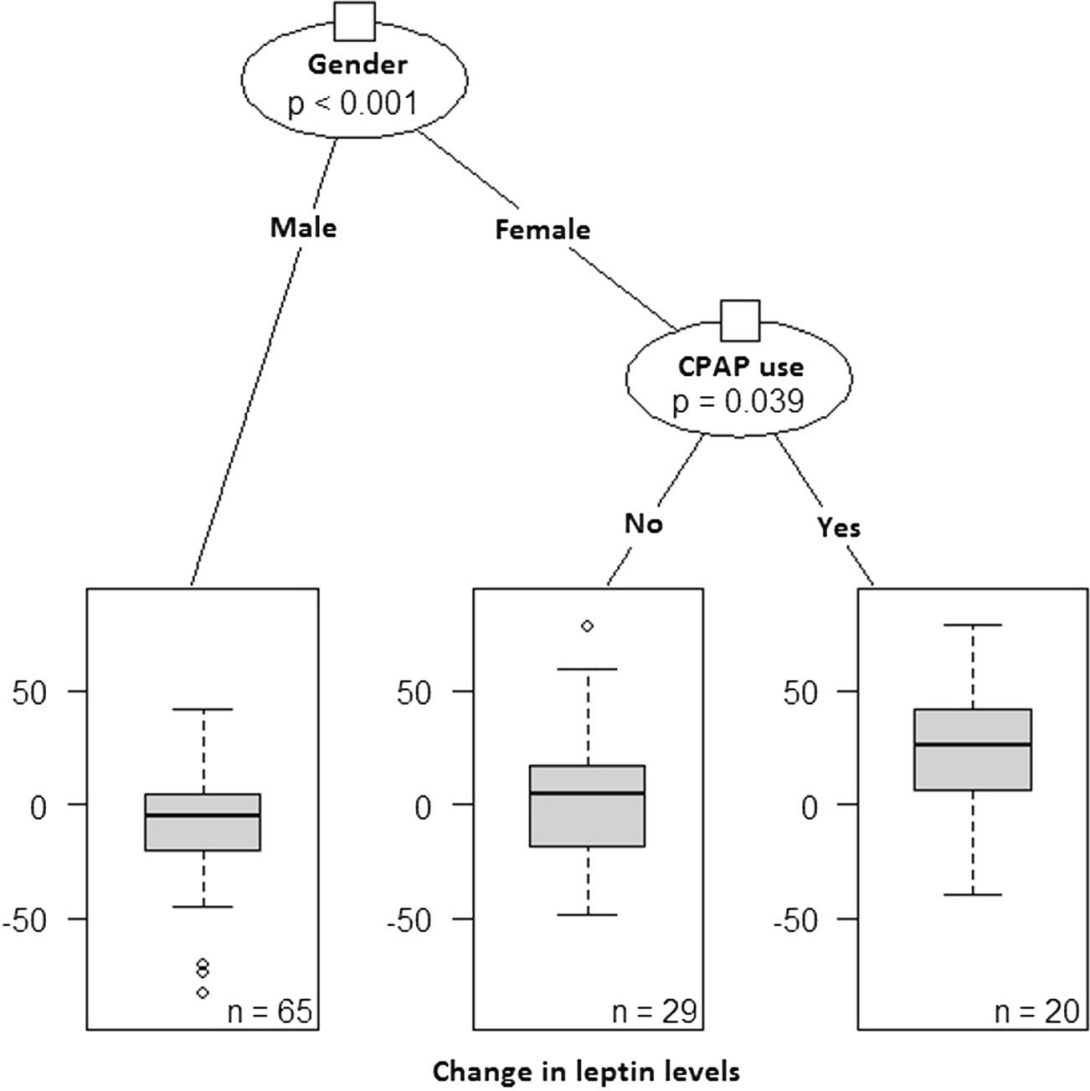
A conditional inference tree visualisation showing the statistically significant interaction between gender and CPAP use. The within-patient 3-year change in leptin levels was greatest for females with CPAP use; whereas for males, the use of CPAP had little effect

At the 3-year follow-up, the CPAP users had higher leptin concentrations (*p* = 0.002) and greater leptin change (*p* = 0.007) than the non-users, and higher BMI (*p* = 0.001), but the change in BMI was not different (Table 2). When males and females were evaluated separately, females had higher leptin concentrations than males (*p* < 0.001), and the change in leptin concentrations was also greater in females than in males (*p* < 0.001) (Table 3, Fig. 2). Leptin concentrations at the 3-year follow-up did not associate with medication defined by the Main Groups of ATC system (data not shown). Further, sleepiness improved in both groups, but the sleepiness improved more among CPAP users (− 3.4 ESS points vs − 2.0 ESS points, *p* = 0.031). Correlation analyses for the array of variables in the entire cohort (Online Resource 1), in males (Online Resource 2), and in females (Online Resource 3) are reported in electronic supplements.

**Table 3:** Differences between the genders at follow-up (*n* = 109)

	Male (*n* = 62)		Female (*n* = 47)		
	Median	IQR (25–75%)	Median	IQR (25–75%)	*p* value
Follow-up BMI (kg/m^2^)	31.07	27.46–35.05	32.47	27.12–37.12	0.338
Change in BMI (kg/m^2^)	0.03	− 0.97–1.29	0.33	− 0.98–2.06	0.491
Follow-up leptin (ng/ml)	7.20	4.31–14.49	32.40	15.64–56.95	*< 0.001*
Change in leptin (ng/ml)	− 3.96	− 22.89–8.23	13.74	− 8.09–35.24	*< 0.001*
Follow-up IGF-1 (nmol/l)	16.45	12.15–19.00	13.90	12.25–19.45	0.317
Change in IGF-1 (nmol/l)	1.65	− 3.23–7.18	2.80	− 2.20–7.09	0.752

Values are presented as median (interquartile range). *BMI* body mass index, *IGF-1* insulin-like growth factor-1, Statistically significant values are shown in italics

**Fig. 3:**
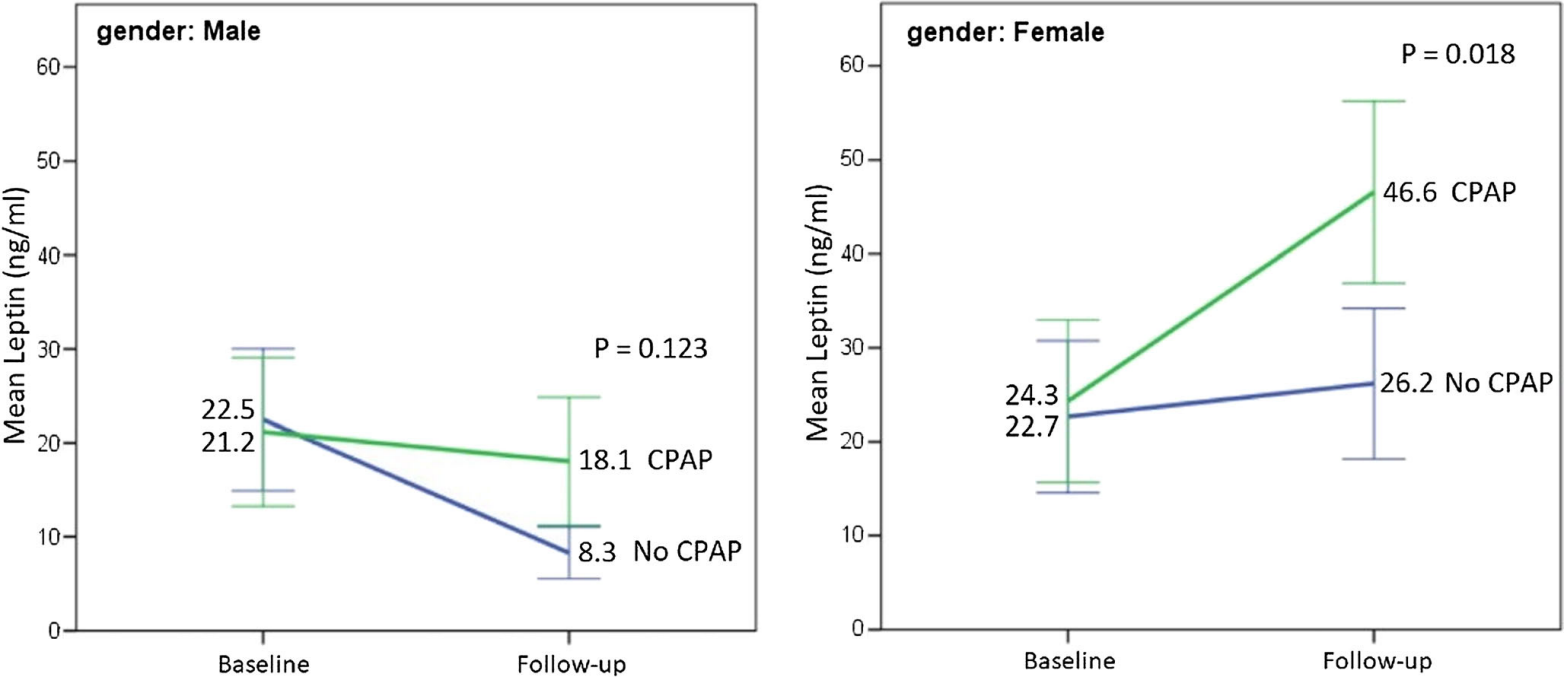
Mean of leptin levels per gender and CPAP use at baseline and follow-up. Whiskers are 95% confidence intervals

Leptin concentrations increased (mean increase 9.13 ng/ml) among the CPAP users compared with the non-users (mean reduction − 5.86 ng/ml) during the follow-up (*p* = 0.007) (Table 2, Fig. 3). The median increase in leptin among female CPAP users was 25.97 ng/ml, and 4.97 ng/ml in the non-users (*p* = 0.010). In males, the change in leptin concentrations did not differ between the CPAP users and non-users (− 0.94 ng/ml vs. − 8.88 ng/ml; *p* = 0.087) (Table 4, Fig. 3). Change in self-reported sleep duration did not differ between the CPAP users and non-users (0.24 h/night vs. 0.09 h/night, *p* = 0.881). At 3-year follow-up, IGF-1 concentrations did not differ between the users and the non-users (Table 2). Weight gain was associated with higher compliance of CPAP use (*r* = 0.290, *p* = 0.046), but not when males (*r* = 0.141, *p* = 0.464) and females (*r* = 0.312, *p* = 0.194) were evaluated separately. Median compliance among the whole group was 6.5 h per night, among males 6.4 h per night, and 6.6 h per night among females (*p* = 0.797).

**Table 4:** Change in leptin concentrations (*n* = 109)

	Male (*n* = 62)			Female (*n* = 47)		
	Median	IQR (25–75%)	*p* value	Median	IQR (25–75%)	*p* value
CPAP users	− 0.94	− 17.43–13.75		25.97	6.22–43.36	
Non-users	− 8.88	− 24.44–0.66	0.087	4.97	− 18.46–17.50	*0.010*

Values are presented as median (interquartile range), Statistically significant values are shown in italics

### Regression analyses

In generalized linear model analysis, change in leptin concentrations and using CPAP did not correlate (*p* = 0.058) among the entire study population, but when males and females were treated separately, the CPAP use was associated with higher leptin concentrations among females (*p* = 0.038) but not in males (*p* = 0.481) (Table 5). The effect between gender and CPAP use on change in leptin concentrations is depicted in Figs. 2 and 3. When the change in BMI was also considered, CPAP use was still a significant factor for change in leptin concentrations. An increase in BMI by 1 kg/m^2^ linked with an increase of 2.1 ng/ml in leptin concentrations (*p* = 0.047, 95% CI 0.03 to 4.2), and CPAP use linked with an increase of 13.0 ng/ml (*p* = 0.017, 95% CI 2.4 to 23.6) in leptin concentrations, based on the ordinary least squares linear regression model.

**Table 5:** Regression analysis for change in leptin concentrations during the 3-year follow-up

		All (*n* = 109)			Male (*n* = 62)			Female (*n* = 47)	
	*B*	95% CI	*p* value	*B*	95% CI	*p* value	*B*	95% CI	*p* value
Gender	27.0	18.41–35.62	*< 0.001*						
CPAP use	7.4	− 0.26–15.09	0.058	− 2.86	− 11.11–5.38	0.481	20.57	1.23–39.92	*0.038*
BMI baseline (kg/m^2^)	1.35	0.64–2.07	*< 0.001*	1.27	0.57–1.97	*< 0.001*	1.59	0.12–3.05	*0.035*
AHI baseline (no./h)	0.22	− 0.17–0.61	0.257	0.25	− 0.35–0.86	0.390	0.12	− 0.61–0.86	0.726
ODI_4_ baseline (no./h)	− 0.14	− 0.52–0.24	0.450	− 0.33	− 0.93–0.27	0.277	− 0.25	− 0.10–0.51	0.500
Median SaO_2_ baseline (%)	− 1.02	− 3.68–1.63	0.442	− 0.28	− 2.53–1.95	0.793	− 3.51	− 10.10–3.07	0.276
Min SaO_2_ baseline (%)	0.55	− 0.30–1.40	0.201	− 0.38	− 1.34–0.59	0.429	0.74	− 1.06–2.54	0.398
Leptin baseline (ng/ml)	− 0.96	− 1.12–0.80	*< 0.001*	− 1.01	− 1.13–0.88	*< 0.001*	− 1.05	− 1.53–0.57	*< 0.001*
IGF-1 baseline (nmol/l)	0.34	− 0.18–0.87	0.193	− 0.07	− 0.54–0.39	0.747	0.26	− 1.00–1.52	0.672
Smoking baseline	− 2.77	− 9.51–3.96	0.412	− 2.88	− 8.64–2.87	0.313	6.01	− 10.82–22.84	0.461
Alcohol baseline	1.24	− 4.55–7.03	0.670	− 1.89	− 6.26–2.49	0.383	15.4	− 0.80–31.5	0.061

*BMI* body mass index, *AHI* apnoea–hypopnoea index, *ODI*_*4*_ oxygen desaturation index, *SaO*_*2*_ arterial oxyhaemoglobin saturation, *IGF-1* insulin-like growth factor-1, Statistically significant values are shown in italics

## Discussion

The major finding in this prospective observational follow-up study was that in patients with OSAS, 3 years of CPAP therapy was associated with increased leptin concentrations in females. Leptin increased independently of age or change in BMI. There was an interaction between leptin increase and gender: leptin increased in female CPAP users; whereas in men, CPAP did not have an effect, if not opposed the natural decrease in leptin observed in men not using CPAP. This finding is contrary to most previous short-term studies, showing that leptin concentrations decrease or remain unchanged in OSAS patients treated with CPAP [18], suggesting that leptin concentrations may behave differently during long-term CPAP treatment, especially among females. The participation of female patients in previous studies has been scarce, making our study essential in indicating the gender-specific difference in leptin concentrations in long-term CPAP therapy. Further, against our hypothesis, among CPAP users, weight gain was associated with higher compliance to CPAP therapy.

Decrease in leptin concentrations has been suggested as an indicator of successful CPAP treatment [19]. Most of the previous studies have had follow-up times less than a year and have reported decrease in leptin concentrations among CPAP users [9, 20–23]. However, there are some studies, which have reported controversial outcomes. Drummond et al. [20] suggested that OSAS does not influence on leptin concentrations. Garcia et al. [21] reported that leptin concentrations remained stable, if the follow-up time was extended into a year. However, their study consisted mainly of men; among the 20 participants, there were only three women. The only two placebo-controlled randomised controlled trials reported no change in leptin concentrations between active and placebo CPAP conditions after 2 [22] or 3 months [23] of treatment. Sanner [15] suggested that CPAP treatment lowers leptin concentrations only if it is efficient, which was documented with polysomnography showing AHI ≤ 5/h with CPAP after 6 months. In their study, leptin concentrations decreased in effectively treated patients, while they increased in ineffectively treated patients. In our study, the median CPAP usage was more than 6 h per night. At follow-up, we neither controlled the effectiveness of treatment with a sleep study, nor did the CPAP devices enable downloading the residual AHI. Therefore, we cannot rule out the possibility of suboptimal therapeutic pressure. However, the CPAP treatment alleviated daytime sleepiness, suggesting that CPAP therapy was effective.

Leptin concentrations in women are higher than in men [24]. In a cross-sectional study in severely untreated OSAS patients using single leptin measurements, OSAS severity was related to leptin concentrations among females, but not in males [25]. In our study, leptin concentrations did not differ between males and females at baseline; whereas during the follow-up, increase in leptin concentrations was higher among females compared with males. Moreover, leptin concentrations were not associated with any medication. Medications were analysed based on ATC Main Groups, because the sample size did not allow more detailed analyses.

The rise in leptin concentrations during long-term CPAP treatment could be explained by several factors. Firstly, short sleep duration is linked with decreased leptin concentrations [26]. When OSAS is treated with CPAP, sleep duration may increase, which however, was not the case in our study. Secondly, sleep timing might change during the follow-up period and affect leptin concentration zenith and nadir times [27]. In our study, nocturnal sleep timing did not change (data not shown). Finally, obese people usually have leptin resistance [13], and patients with OSAS are mainly obese. Usually, elevated leptin concentrations result in reduction in appetite [7], but leptin resistance prevents this [28]. Leptin resistance might explain at least in partly, why most patients do not lose weight during long-term CPAP therapy [5].

IGF-1 concentrations did not change during the 3-year follow-up. According to a recent meta-analysis, the effect of CPAP therapy on IGF-1 concentrations in patients with OSAS is still controversial [9]. Münzer et al. [29] reported that IGF-1 concentrations were increased in males aged 40–60 only. In females and older men (over 60 years), the IGF-1 concentrations did not increase during 8 months of CPAP treatment. Our study was gender-balanced and had more females than most studies, which could explain why IGF-1 concentrations remained unchanged. Median age of our patients was 54 years, which is in line with previous studies.

Contrary to our hypothesis, weight gain was not associated with OSAS severity. This is in line with a recent study, where 80% of patients with CPAP-treated OSAS showed no significant change in weight over a follow-up period of 7 years [5]. Moreover, contrary to our expectations, weight gain among CPAP users was associated with higher compliance. This may be explained by reduced basal metabolic rate or decreased sleep energy expenditure during CPAP treatment resulting in weight gain [6]. However, when males and females were evaluated separately, the association disappeared.

The strength of our study is a longer follow-up time than in previous studies [9, 18]. Our cohort was gender-balanced, and of moderate size, whereas previous studies have included mainly males, and their sample sizes have been significantly smaller, with the exception of one 1-month study [20]. Some limitations should be considered when interpreting our results. We had only single measurements of leptin concentrations, although leptin is known to have a circadian rhythm. However, leptin concentrations were measured approximately at the same clock time at baseline and follow-up. Further, we did not measure leptin concentrations shortly after the initiation of the CPAP treatment, and therefore, we cannot know, whether leptin concentrations increased already in the early stage of the CPAP treatment, or if the concentrations increased later, but this does not compromise our finding of leptin increase after 3 years of CPAP use.

## Conclusion

Our study suggests that long-term CPAP treatment elevates leptin concentrations in female patients with OSAS, independently of age or BMI. This finding is in contrary to earlier short-term studies and suggests that leptin concentrations may behave differently during long-term CPAP therapy. Our results need to be interpreted cautiously, since the sample size was moderate, and we did not have repetitive measurements during the 3-year follow-up, to show whether the leptin concentrations remained at the steady level over the follow-up period. Further prospective studies with repetitive leptin measurements over long-term CPAP treatment are warranted, to achieve a deeper insight into the metabolic regulation of OSAS over the course of CPAP treatment.

## Electronic supplementary material

ESM 1 (DOCX 18 kb)

ESM 2 (DOCX 15 kb)

ESM 3 (DOCX 15 kb)
