# Supplementary material for: Gender-specific change in leptin concentrations during long-term CPAP therapy
Source: Sleep Breath. 2019 May 4;24(1):191–9. doi: 10.1007/s11325-019-01846-y (PMC7128000; doi:10.1007/s11325-019-01846-y)
Supplement: Supplementary file 1 — (DOCX 18 kb) [file 11325_2019_1846_MOESM1_ESM.docx]

|  | **Gender** | **CPAP use** | **Baseline BMI** (kg/m^2^) | **Follow-up BMI** (kg/m^2^) | **Change in BMI** (kg/m^2^) | **Baseline leptin** (ng/ml) | **Follow-up leptin** (ng/ml) | **Change in leptin levels** (ng/ml) |
| --- | --- | --- | --- | --- | --- | --- | --- | --- |
| **Baseline age** (years) | p= 0.809  r= -0.023 | p= 0.664  r=-0.042 | p= 0.524  r=0.060 | p= 0.340  r=0.090 | p= 0.493  r=0.065 | p= 0.437  r=0.075 | **p<0.001**  r=0.529 | p=0.235  r=0.112 |
| **CPAP use** | p= 0.664  r= -0.042 |  | **p<0.001**  r= 0.351 | **p<0.001**  r= 0.365 | p= 0.252  r= 0.111 | p= 0.871  r= 0.016 | **p= 0.001**  r= 0.310 | **p= 0.007**  r= 0.258 |
| **Baseline BMI**(kg/m^2^) | p= 0.524  r=0.060 | **p<0.001**  r=0.351 |  | **p<0.001**  r=0.918 | p=0.295  r=-0.099 | p=0.259  r=-0.109 | **p<0.001**  r=0.619 | **p<0.001**  r=0.478 |
| **Follow-up BMI**(kg/m^2^) | p= 0.340  r=0.090 | **p<0.001**  r=0.365 | **p<0.001**  r=0.918 |  | **p= 0.006**  r=0.255 | p= 0.457  r=-0.072 | **p<0.001**  r=0.731 | **p<0.001**  r=0.537 |
| **Change in BMI**(kg/m^2^) | p= 0.493  r=0.065 | p= 0.252  r=0.111 | p=0.295  r=-0.099 | **p=0.006**  r=0.255 |  | p= 0.925  r=0.009 | **p<0.001**  r=0.323 | **p= 0.016**  r=0.227 |
| **Baseline**  **AHI**(#/h) | p= 0.521  r=-0.062 | **p<0.001**  r=0.511 | **p<0.001**  r=0.417 | **p<0.001**  r=0 .347 | p= 0.283  r=-0.104 | p= 0.771  r=-0.029 | **p= 0.014**  r=0.241 | **p= 0.017**  r=0.229 |
| **Baseline**  **ODI_4_** (#/h) | p= 0.965  r=-0.004 | **p<0.001**  r=0.486 | **p<0.001**  r=0.559 | **p<0.001**  r=0.518 | p= 0.981  r=-0.002 | p= 0.762  r=-0.030 | **p<0.001**  r=0.368 | **p<0.001**  r=0.308 |
| **Mean SaO_2_ baseline**(%) | p=0.122  r=0.146 | **p<0.001**  r=-0.354 | **p<0.001**  r=-0.559 | **p <0.001**  r=-0.536 | p=0.965  r=0.004 | p=0.201  r=0.124 | **p<0.001**  r=-0.339 | **p<0.001**  r=-0.338 |
| **MinSaO_2_ baseline**(%) | p=0.401  r=-0.080 | **p<0.001**  r=-0.335 | **p<0.001**  r=-0.541 | **p<0.001**  r=-0.505 | p=0.863  r=-0.016 | p=0.792  r=0.026 | **p<0.001**  r=-0.450 | p<0.001  r=-0.353 |
| **Baseline leptin** (ng/ml) | p=0.437  r=0.075 | p=0.871  r=0.016 | p=0.259  r=-0.110 | p=0.457  r=-0.072 | p=0.924  r=0.009 |  | p=0.952  r=-0.006 | **p<0.001**  r=-0.607 |
| **Follow-up leptin** (ng/ml) | **p<0.001**  r=0.529 | **p=0.001**  r=0.310 | **p<0.001**  r=0 .619 | **p<0.001**  r=0.731 | **p<0.001**  r=0.323 | p=0.953  r=-0.006 |  | **p<0.001**  r=0 .713 |
| **Change in leptin levels** (ng/ml*)* | **p<0.001**  r=0.361 | **p=0.007**  r=0.258 | **p <0.001**  r=0.478 | **p<0.001**  r=0.537 | **p=0.016**  r=0.227 | **p<0.001**  r=-0.607 | **p<0.001**  r=0.713 |  |
| **Baseline**  **IGF-1** (nmol/l) | **p=0.018**  r=-0.224 | p= 0.596  r=0.052 | p= 0.974  r=0.003 | p= 0.971  r=-0.003 | p= 0.417  r=-0.078 | p= 0.134  r=-0.146 | p= 0.676  r=-0.041 | p=0.359  r=0.088 |
| **Follow-up IGF-1** (nmol/l) | p= 0.319  r=-0.096 | p= 0.934  r=-0.008 | p=0.183  r=-0.129 | p=0.140  r=-0.142 | p=0.793  r=0.025 | p= 0.425  r=0.079 | p=0.189  r=-0.129 | **p= 0.049**  r=-0.188 |
| **Change in IGF-1** (nmol/l) | p=0.759  r=0.030 | p=0.777  r=-0.028 | p=0.439  r=0.440 | p=0.358  r=-0.090 | p=0.615  r=0.050 | p=0.254  r=0.114 | p=0.376  r=-0.089 | **p=0.038**  r=-0.201 |

Online resource 1. Correlations between the variables in the entire cohort. BMI body mass index, AHI apnoea-hypopnoea index, ODI_4_ oxygen desaturation index, SaO_2_ arterial oxyhaemoglobin saturation, IGF-1 insulin-like growth factor-1.

Gender-specific increase in leptin concentrations during long-term CPAP therapy

Sleep and Breathing. Aro Miia MD, Division of Medicine, Department of Pulmonary Diseases, Turku University Hospital, Turku, Finland email: miia.aro@tyks.fi.
