# Supplementary material for: Gender-specific change in leptin concentrations during long-term CPAP therapy
Source: Sleep Breath. 2019 May 4;24(1):191–9. doi: 10.1007/s11325-019-01846-y (PMC7128000; doi:10.1007/s11325-019-01846-y)
Supplement: Supplementary file 2 — (DOCX 15 kb) [file 11325_2019_1846_MOESM2_ESM.docx]

|  | **CPAP use** | **Baseline BMI** (kg/m^2^) | **Follow-up BMI** (kg/m^2^) | **Change in BMI** (kg/m^2^) | **Baseline leptin** (ng/ml) | **Follow-up leptin** (ng/ml) | **Change in leptin levels** (ng/ml) |
| --- | --- | --- | --- | --- | --- | --- | --- |
| **Baseline age** | p=0.955  r=-0.007 | p=0.759  r=-0.039 | p=0.469  r=-0.091 | p=0.179  r=-0.170 | p=0.299  r=-0.133 | p=0.852  r= 0.025 | p=0.165  r= 0.174 |
| **CPAP use** |  | **p=0.007**  r=0.340 | **p=0.026**  r= 0.283 | p=0.743  r=0.043 | p=0.810  r=-0.032 | **p=0.008**  r=0.350 | p=0.087  r=0.219 |
| **Baseline BMI** *(*kg/m^2^) | **p=0.007**  r=0.340 |  | **p<0.001**  r=0.917 | p=0.876  r=0.020 | p=0.505  r=-0.086 | **p<0.001**  r=0.792 | **p<0.001**  r=0.453 |
| **Follow-up BMI** (kg/m^2^) | **p=0.026**  r=0.283 | **p<0.001**  r=0.917 |  | **p=0.002**  r=0.373 | p=0.470  r=-0.093 | **p<0.001**  r=0.868 | **p<0.001**  r=0.533 |
| **Change in BMI** (kg/m^2^*)* | p=0.743  r=0.043 | p=0.877  r=0.020 | **p=0.002**  r=0.373 |  | p=0.177  r=-0.174 | **p=0.002**  r=0.382 | **p<0.001**  r=0.421 |
| **Baseline AHI** (*#/h)* | **p<0.001**  r=0.530 | **p=0.002**  r=0.381 | **p=0.032**  r=0.495 | p=0.908  r=0.015 | p=0.757  r=0.041 | p=0.059  r=0.256 | p=0.460  r=0.097 |
| **Baseline ODI_4_** (*#/h)* | **p<0.001**  r=0*.481* | **p<0.001**  r=0.560 | **p<0.001**  r=0*.*495 | p=0.908  r=0.015 | p=0.730  r=0.045 | **p=0.003**  r=0.384 | p=0.154  r=0*.*181 |
| **Baseline SaO_2_ mean** (%) | **p*=*0.024**  r=-0*.*288 | **p<0.001**  r=-0.525 | **p<0.001**  r=-0.521 | p=0.230  r=-0.152 | p=0.530  r= 0.081 | **p<0.001**  r=-0.538 | **p=0.001**  r=-0*.*396 |
| **Baseline SaO_2_ min** (%) | **p=0.005**  r=-0.353 | **p<0.001**  r=-0.489 | **p<0.001**  r=-0.481 | p=0.215  r=-0.157 | p=0.683  r=-0.053 | **p<0.001**  r=-0.480 | **p*=*0.038**  r=-0.260 |
| **Baseline leptin** (ng/ml) | p=0.810  r=-0.032 | p=0.505  r=-0.086 | p=0.470  r=-0.093 | p=0.177  r=-0.174 |  | p=0.374  r=-0.119 | **p<0.001**  r=-0.761 |
| **Follow-up leptin** (ng/ml) | **p=0.008**  r=0.350 | **p<0.001**  r=0.792 | **p<0.001**  r=0.868 | **p=0.002**  r=0.382 | p=0.374  r=-0.119 |  | **p<0.001**  r=0.615 |
| **Change in leptin levels** (ng/ml) | p=0.087  r=0.219 | **p<0.001**  r=0.453 | **p<0.001**  r=0.533 | **p=0.001**  r=0.421 | **p<0.001**  r=-0.761 | **p<0.001**  r=0.615 |  |
| **Baseline IGF-1** (nmol/l) | p=0.253  r=-0.149 | p=0.691  r=-0.051 | p=0.779  r=-0.036 | p=0.590  r=-0.069 | p=0.813  r=-0.031 | p=0.627  r=0.065 | p=0.977  r=0.004 |
| **Follow-up IGF-1** (nmol/l) | p=0.878  r=-0.020 | p=0.420  r=0.104 | p=0.990  r=-0.002 | p=0.555  r=-0.076 | p=0.868  r= 0.022 | p=0.891  r=0.018 | p=0.580  r=-0.071 |
| **Change in IGF-1** (nmol/l) | p=0.657  r=0.058 | p=0.315  r=0.131 | p=0.775  r=0.037 | p=0.983  r=0.003 | p=0.804  r=-0.033 | p=0.912  r=0.015 | p=0.949  r=-0.008 |

Online resource 2. Correlations between variables in males (n=65). BMI body mass index, AHI apnoea-hypopnoea index, ODI_4_ oxygen desaturation index, SaO_2_ arterial oxyhaemoglobin saturation, IGF-1 insulin-like growth factor-1.

Gender-specific increase in leptin concentrations during long-term CPAP therapy

Sleep and Breathing. Aro Miia MD, Division of Medicine, Department of Pulmonary Diseases, Turku University Hospital, Turku, Finland email: miia.aro@tyks.fi.
