# Supplementary material for: Gender-specific change in leptin concentrations during long-term CPAP therapy
Source: Sleep Breath. 2019 May 4;24(1):191–9. doi: 10.1007/s11325-019-01846-y (PMC7128000; doi:10.1007/s11325-019-01846-y)
Supplement: Supplementary file 3 — (DOCX 15 kb) [file 11325_2019_1846_MOESM3_ESM.docx]

|  | **CPAP use** | **Baseline BMI** (kg/m^2^) | **Follow-up BMI** (kg/m^2^) | **Change in BMI** (kg/m^2^*)* | **Baseline leptin** (ng/ml) | **Follow-up leptin** (ng/ml) | **Change in leptin levels** (ng/ml) |
| --- | --- | --- | --- | --- | --- | --- | --- |
| **Baseline age** (years) | p=0.327  r= 0.146 | p=0.227  r=-0.176 | p=0.354  r=-0.135 | p=0.359  r=0.134 | p=1.000  r=-0.000 | p=0.839  r=0.030 | p=0.828  r=0.032 |
| **CPAP use** |  | **p=0.009**  r=0.378 | **p<0.001**  r=0.489 | p=0.185  r=0.197 | p=0.526  r=0.098 | **p=0.001**  r=0.460 | **p=0.009**  r=0.378 |
| **Baseline BMI** (kg/m^2^*)* | **p=0.008**  r=0.378 |  | **p<0.001**  r=0.907 | p=0.091  r=-0.244 | p=0.273  r=-0.165 | **p<0.001**  r=0.653 | **p<0.001**  r=0.561 |
| **Follow-up BMI** *(*kg/m^2^) | **p<0.001**  r=0.489 | **p<0.001**  r=0.907 |  | p=0.445  r=0.112 | p=0.859  r=*-*0.027 | **p<0.001**  r=0.800 | **p<0.001**  r=0.584 |
| **Change in BMI** (kg/m^2^*)* | p=0.185  r=0.197 | p=0.091  r=-0.244 | p=0.445  r=0.112 |  | p=0.110  r=0.239 | **p=0.037**  r=0.299 | p= 0*.*750  r=0.047 |
| **Baseline AHI** (#/h) | **p<0.001**  r= 0.499 | **p=0.001**  r= 0.477 | **p=0.002**  r=0.442 | p=0.755  r=-0.046 | p=0.408  r=-0.126 | **p=0.005**  r= 0.395 | **p=0.002**  r=0.427 |
| **Baseline ODI_4_** (#/h*)* | **p<0.001**  r=0.491 | **p<0.001**  r=0.599 | **p<0.001**  r=0.566 | p=0.778  r=-0.042 | p=0.357  r=*-*0.140 | **p<0.001**  r=0.499 | **p<0.001**  r=0.468 |
| **Baseline SaO_2_ mean** (%) | **p=0.003**  r=-0.422 | **p<0.001**  r=-0.640 | **p<0.001**  r=-0.605 | p=0.388  r=0.126 | p= 0.366  r=0.136 | **p<0.001**  r=-0.487 | **p*=*0.003**  r=-0.417 |
| **Baseline SaO_2_ min** (%) | **p=0.015**  r=-0.422 | **p<0.001**  r=-0.64 | **p<0.001**  r=-0.605 | p=0.388  r=0.126 | p=0.366  r=0.136 | **p<0.001**  r=-0.487 | **p=0.010**  r=-0.366 |
| **Baseline leptin** (ng/ml) | p=0.526  r= 0.099 | p=0.273  r=-0.165 | p=0.859  r=0.489 | p=0.185  r=0.197 |  | p=0.832  r=-0.032 | **p<0.001**  r=-0.605 |
| **Follow-up leptin** (ng/ml) | **p=0.001**  r=0.460 | **p<0.001**  r=0.653 | **p<0.001**  r=0.800 | **p=0.037**  r=0.299 | p=0.832  r=-0.032 |  | **p<0.001**  r=0.778 |
| **Change in leptin levels** (ng/ml) | **p= 0.009**  r=0.378 | **p<0.001**  r=0.561 | **p<0.001**  r=0.584 | p= 0.751  r=0.047 | **p<0.001**  r=-0.605 | **p<0.001**  r=0.778 |  |
| **Baseline IGF-1** (nmol/l) | **p=0.048**  r=0.296 | p=0.490  r=0.103 | p=0.644  r=0.069 | p=0.497  r=-0.102 | p=0.062  r=-0.284 | p=0.410  r=0.123 | **p= 0.014**  r=0.354 |
| **Follow-up IGF-1**  (nmol/l) | p=0.973  r=0.005 | **p=0.009**  r=-0.378 | **p=0.036**  r=-0.307 | p=0.381  r=0.131 | p=0.285  r=0.165 | p=0.097  r=-0.245 | p=0.053  r=-0.284 |
| **Change in IGF-1 levels** (nmol/l) | p=0.374  r=-0.139 | p=0.055  r=-0.288 | p=0.123  r=-0.233 | p=0.438  r=0.118 | **p=0.042**  r=0.316 | p=0.066  r=-0.276 | **p=0.001**  r=-0.468 |

Online resource 3. Correlations between variables among women (n=49). BMI body mass index, AHI apnoea-hypopnoea index, ODI_4_ oxygen desaturation index, SaO_2_ arterial oxyhaemoglobin saturation, IGF-1 insulin-like growth factor-1.

Gender-specific increase in leptin concentrations during long-term CPAP therapy

Sleep and Breathing. Aro Miia MD, Division of Medicine, Department of Pulmonary Diseases, Turku University Hospital, Turku, Finland email: miia.aro@tyks.fi.
